# How Do Clinicians Learn About Knowledge Translation? An Investigation of Current Web-Based Learning Opportunities

**DOI:** 10.2196/mededu.7825

**Published:** 2017-07-13

**Authors:** Raechel A Damarell, Jennifer J Tieman

**Affiliations:** ^1^ Palliative and Supportive Services Flinders University Adelaide SA Australia

**Keywords:** knowledge, translational medical research, diffusion of innovation, health personnel, education, medical, continuing, quality assurance, health care

## Abstract

**Background:**

Clinicians are important stakeholders in the translation of well-designed research evidence into clinical practice for optimal patient care. However, the application of knowledge translation (KT) theories and processes may present conceptual and practical challenges for clinicians. Online learning platforms are an effective means of delivering KT education, providing an interactive, time-efficient, and affordable alternative to face-to-face education programs.

**Objective:**

This study investigates the availability and accessibility of online KT learning opportunities for health professionals. It also provides an analysis of the types of resources and associated disciplines retrieved by a range of KT synonyms.

**Methods:**

We searched a range of bibliographic databases and the Internet (Google advanced option) using 9 KT terms to identify online KT learning resources. To be eligible, resources had to be free, aimed at clinicians, educational in intent, and interactive in design. Each term was searched using two different search engines. The details of the first 100 websites captured per browser (ie, n=200 results per term) were entered into EndNote. Each site was subsequently visited to determine its status as a learning resource. Eligible websites were appraised for quality using the AACODS (Authority, Accuracy, Coverage, Objectivity, Date, Significance) tool.

**Results:**

We identified 971 unique websites via our multiple search strategies. Of these, 43 were health-related and educational in intent. Once these sites were evaluated for interactivity, a single website matched our inclusion criteria (Dementia Knowledge Translation Learning Centre).

**Conclusions:**

KT is an important but complex system of processes. These processes overlap with knowledge, practice, and improvement processes that go by a range of different names. For clinicians to be informed and competent in KT, they require better access to free learning opportunities. These resources should be designed from the viewpoint of the clinician, presenting KT’s multifaceted theories and processes in an engaging, interactive way. This learning should empower clinicians to contextualize and apply KT strategies within their own care settings.

## Introduction

Efforts to improve the quality of health care delivery for better patient outcomes continue to be impeded by a gap between the creation and dissemination of high quality evidence and its translation into clinical practice and policy. We know this disconnect can result in under-prescription of proven, effective treatments, or the continued promotion of less effective or even harmful interventions [1]. It also contributes to wastage of finite health care resources [2,3] and an unacceptable lag time in getting mass recognition of what constitutes best practice [4].

The emergence of the evidence-based medicine (EBM) paradigm in the 1970s focused attention on the need for individual clinicians to seek, appraise, and judiciously apply research evidence in tandem with their own clinical judgment and patient preferences [5]. While EBM has inevitably resulted in a more conscientious seeking of evidence by clinicians with concomitant benefits for patients, its focus is squarely on individual clinician decision-making responsibilities. It does not, and cannot, address the levers, mechanisms, and barriers that effect systematic and sustainable change within the complex organizations and systems in which clinicians work. These concerns are rather the chief domain of an emerging area of research—knowledge translation (KT or “implementation science”).

### What Is KT and How Can It Help?

KT has emerged as an interdisciplinary field of research and practice to address the gap between what is known to work and what is done in practice [6]. The Canadian Institutes of Health Research (CIHR) provides the most commonly cited formal definition of KT as “a dynamic and iterative process that includes the synthesis, dissemination, exchange and ethically sound application of knowledge to improve health, provide more effective health services and products and strengthen the health care system” [7]. KT practice, as distinct from KT research, is concerned with helping knowledge stakeholders—clinicians, patients, health system managers and administrators, and decision makers—become aware of knowledge and facilitate “use of it in their day-to-day work and decision making” [8].

While KT practice interacts with a range of activities, including EBM, continuing medical education, continuing professional development, and quality improvement, it is much broader than all of these [9]. Its focus is on developing and evaluating interventions capable of bringing about practice change in real world settings, providing evidence of which strategies work and which do not, as well as practical guidance on how these strategies can be used to drive change across health care settings.

### Challenges With KT

A number of KT characteristics may present conceptual and practical challenges for the clinician-learner. First, KT does not provide a linear, or even systematic equation for effecting change. It involves complex, multi-dimensional processes incorporating the values, knowledge, and behaviors of individuals from different professions, organizational priorities, and perhaps even conflicting political agendas. Second, multiple theoretical models have emerged in an attempt to reduce the complexity surrounding KT and provide a coherent overarching framework for KT practice [10-13]. This lack of a single, unifying theory may be confusing for would-be KT practitioners.

KT also suffers from a lack of conceptual clarity with many terms competing to describe all or parts of its remit [1,14]. These terms include *knowledge transfer*, *research utilization, knowledge-to-action, implementation science*, and *diffusion and dissemination*. Some of these (eg, *diffusion and dissemination*) are focused on the researcher’s—rather than the knowledge user’s—perspective. Other terms, such as *knowledge transfer* or *knowledge exchange*, appear to describe the processes for transmitting knowledge from researcher to user, rather than providing clues as to how clinicians may translate knowledge for use. KT as a metaphor has also been questioned for potentially constraining how we conceptualize both “knowledge” and the ways in which it might be “translated” in real world practice [15].

KT uptake may also be impeded by confusion between what could be termed “knowledge” activities (eg, continuing professional development and evidence based practice), and “improvement” activities such as quality improvement and clinical audit. The consequential risk for clinicians is losing sight of the nexus between the knowledge and translation parts of KT.

Another challenge lies in the fact that while myriad types of KT strategies are described in the research literature, many have shown limited efficacy or have been applied or reported in a way that makes them difficult to replicate or even compare with other studies or interventions [16,17].

### KT Education

If KT is complex and yet an important deliberative approach to improving quality of care, we should expect KT education to be foundational and KT training and capacity building to be currently taking place across all levels of health professional education. We might even expect health care organizations to have developed the infrastructure to support, sustain, and normalize KT activities.

Despite these expectations, and the formalization of KT competencies [18,19], health professional KT education opportunities remain far from ubiquitous [6,8,19]. At the time of developing their own national training initiative in 2011, CIHR could not identify existing national programs on which to model theirs [18]. While training programs are now beginning to emerge, many are only available via competitive application [20], formal university-level courses [21,22], or locally run fee-based workshops. These programs, however, require significant time and monetary costs [6], a reality likely to deter the majority of clinicians from engaging in KT education.

Other initiatives are evolving to address this limitation. These include local mentoring programs [23], short-term, contextualized, multidisciplinary team projects focused on a single area of care [24], and online KT communities of practice [8,25]. Educators are also suggesting innovative ways for KT education to be integrated into health professional education curricula [26,27].

### What Do Clinicians Want?

Several studies have used qualitative methods to determine clinician understanding of and interest in KT [6,23,28,29]. The Holmes study found that clinicians have a strong desire to learn more about KT, but 63.03% (675/1071) believe they would require beginner level training. Clinicians also want flexible, easily accessible, and inexpensive training options such as small group learning opportunities or self-guided study. Most significantly, 85.99% (921/1071) reported a willingness to engage with free Web-based training programs [28].

Another study found that many clinicians report a basic understanding of the principles of KT while being unfamiliar with the term itself. These clinicians also believed they lacked the skills to undertake KT projects and cited a preference for interactive, time-efficient, and brief training opportunities [23]. A further study by Lal [6] highlighted KT-specific learning challenges such as scarce training resources and practitioner difficulties in adapting KT theories to specific clinical settings.

These findings may be indicative of a need for more readily accessible KT training resources at the foundational level. Ideally, such resources would define KT, explain its benefits in terms of patient outcomes, and provide illustrative examples of how specific KT models and strategies might be adapted and applied to local environments.

The high level of clinician interest in freely available online resources for continuing education warrants attention. We know online learning opportunities offer learners control over how, when, and where they interact with learning materials, making it possible to determine the sequence and pace of one’s own learning [30]. Web-based learning can also facilitate self-assessment of competence [31]. For this reason, Web-based learning platforms have become commonplace in postgraduate education provision. One meta-analysis of Web-based learning effectiveness studies found favorable outcomes for this mode of delivery across a range of learning contexts and health and medical topics. These include significant gains in knowledge and flow-on improvements in patient care behaviors [32]. Another synthesis found an association between improved learning outcomes and the degree of resource interactivity, repetition, and feedback, as well as the availability of practice exercises [33]. Other positive outcomes reported in the research literature include improved skills [34,35], higher clinician satisfaction with the online mode over other formats [36], improvements in guideline adherence [37], and increased implementation of knowledge into practice [38]. In this sense, Web-based learning platforms may be regarded as effective KT interventions in their own right.

As part of a project funded by Australia’s National Health and Medical Research Centre, we wish to identify existing high quality online training modules on KT targeted at health professionals. If these modules are suitable, our intention is to use them as a template in developing our own learning module or seek permission to incorporate them into a new suite of learning resources provided on a new Centre for Research Excellence website. However, based on clinician reports in the literature, we anticipate that Web-based opportunities are either scarce or difficult to find.

The main objective of this investigation was therefore to conduct a comprehensive open Web search for online KT learning opportunities available to health professionals. Our goal was to determine whether such opportunities already exist or whether there is a need for resource development in this area.

## Methods

### Resource Selection Criteria

To be eligible for consideration in this review, a Web resource had to be: (1) published in English, (2) freely available online or available via free registration, (3) targeted at health professionals, health researchers, or health students, (4) educational in orientation, meaning its purpose is to develop health professional knowledge of KT in a systematic and incremental way rather than just providing information, and (5) interactive in design.

We defined “Interactive” as meaning end-users engage online with a single standalone resource comprising a mixture of text, images, audio, video, animation, and perhaps even online discussions. Interactive resources require users to work through the materials sequentially, and at their own pace, providing scope for reflection and activities for testing the understanding of the material.

Irrespective of their quality and authoritativeness, static resources such as PDF workbooks and other materials designed to be printed and worked through offline were deemed ineligible for the review due to their lack of interactivity. We also excluded resources for “doing” KT such as toolkits and strategy checklists, as well as didactic PowerPoint presentations, webinars, and resources comprising lists of Web links, unless these resources were part of a broader, cohesive online learning module.

### Search Strategies

One author conducted the searches (RD). These were executed, without date restriction, on July 23, 2015. Searches were limited to English language resources only.

We used multiple approaches to identifying online learning resources. These included:

- Limited searches of databases Medline (Ovid), Embase (Ovid), Scopus, and ERIC (ProQuest) for online KT learning resources named in published research articles. An example of our database search strategy is provided as Multimedia Appendix 1. This strategy was modified for each database.
- A sampling approach to online searching using Web search engine Google (advanced option). A number of variant searches were run in an attempt to overcome limitations on search sensitivity imposed by Web search engines.
- A separate search of MOOC sites, webinars, and YouTube clips.
- A scan of the websites of KT-focused organizations identified in stage two (eg, Canadian Institutes of Health Research) for links to other learning resources not picked up by the Web search itself.

### Term Variants

KT is known by a wide range of terms [39,40]. To ensure we did not overlook any learning modules, we searched on nine of the most prevalent KT terms:

- Knowledge translation
- Knowledge transfer
- Knowledge exchange
- Research utilization
- Research utilisation
- Implementation science
- Research into practice
- Knowledge-to-action
- Evidence-to-use.

Each term was entered on its own in the Google Advanced “this exact word or phrase” search field.

### Search Restrictions

In an attempt to focus the search on sites with educational intent, we added the following search string to each KT term search: *module OR modules OR train OR training OR learn OR learning OR teach OR teaching OR educate OR educating OR education OR educational OR program OR programme OR study OR CME OR CPD.*

We did not include health-related search terms, partly as the limited search features of Google would not allow too many variants at one time. We were also interested to see which health disciplines are associated with KT education efforts.

### Allowance for Web Browser Effects

All 9 KT term variants were first searched using Mozilla Firefox (with behavior tracking), and the first 100 results for each term were copied into a Word document. This process was then repeated using Chrome with incognito browsing functionality in an attempt to maximize the number of unique retrievals across browsers. Incognito browsing disables a computer’s browsing history and Web cache, ensuring websites are retrieved and ranked based on the weighted inclusion of a specific search term within that website, rather than a searcher’s previous browsing activity. We therefore retrieved 200 websites for each of the 9 KT terms searched.

### Data Collection and Analysis

All websites identified by each Google search were manually recorded in an EndNote library. Information captured included website author, title of page, and URL. Duplicate entries (ie, websites identified by more than one search) were identified and removed.

Both investigators (RD and JT) independently screened the same random set of 50 items taken from the full results set in order to test inclusion/exclusion criteria and ensure data extraction requirements had been fully thought through. This involved using the URL recorded to access the webpage and review it for relevance. One investigator then screened and categorized the remaining results with the aid of a research assistant.

For each website retrieved, the following details were entered in customized fields of the EndNote record in the form of a yes or no entry:

- For a health audience?
- Educational in intent?
- Freely available online?
- Interactive in design?
- Defines KT?

### Quality Assessment

Finally, each included resource was assessed for quality using the AACODS checklist for appraising gray literature [41]. This checklist focuses on six domains: authority, accuracy, coverage, objectivity, date, and significance. Online learning modules not meeting the standard set by this checklist were to be excluded.

## Results

The total number of websites retrieved by our multiple search strategies was 1800. This reduced to 971 after duplicate entries were removed. The database searches yielded two reports describing online KT learning resources [20,42]. Both resources were also identified by the Web search.

**Figure 1:**
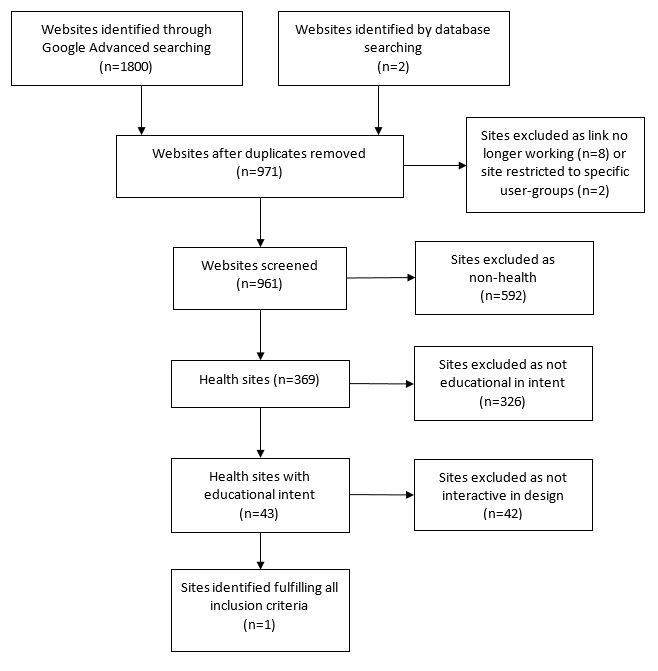
PRISMA flow diagram of selection decisions.

The results of evaluating retrievals against inclusion criteria are shown in the form of a PRISMA (Preferred Reporting Items for Systematic Reviews and Meta-Analyses) flow diagram (Figure 1).

Of the 971 unique websites retrieved, only 43 health-relevant KT websites with educational intent were identified and comprehensively reviewed. Resources were categorized as educational if they contained explicit statements of learning objectives and provided, as a minimum, a basic definition of KT. A breakdown of the types of resources fulfilling these criteria is shown as part of Table 1.

These 43 sites were then judged on the “interactivity” of their design. At this point, 42 of the 43 resources were eliminated on the basis that they comprised a list of resources, or links to resources, without an overarching instructional framework, or provided KT learning materials in the form of non-integrated, non-sequential informational webpages or documents.

Only one resource met all our inclusion criteria and could be designated an online, self-paced learning module on KT for health professionals. This resource was the Dementia Knowledge Translation (DKT) Learning Centre by Canadian Dementia Knowledge Translation Network [42].

The self-described purpose of the DKT Learning Centre is to enable researchers to “learn more about how to conduct and adapt dementia studies to inform further research, and to ultimately use the new knowledge gained to improve the treatment and care of persons with dementia and support their caregivers” [43]. The rationale for this free resource came from a 2011 Web-based survey of Canadian dementia researchers [44]. This survey revealed a high level of practitioner interest in translating dementia knowledge and was instrumental in identifying specific training needs and priorities. There was particular interest in self-paced training programs offered over the Internet.

The DKT Learning Centre presents KT under four broad headings: (1) introduction to KT, (2) what is Dementia KT?, (3) DKT in grants, and (4) DKT dissemination & exchange. Standard sections beneath these headings included “learning objectives,” ”discuss this topic,” and “evaluate.” The resource provides access to a wide range of resources such as further readings, dementia KT examples, and sample budgets. We judged it to be of high quality using AACODS. This was based on its: (1) authoritative authorship, (2) accuracy (states and meets it aims and is well referenced), (3) coverage (has clear parameters), (4) objectivity, (5) date, and (6) significance (adds value in terms of utility and relevance).

**Table 1:** Knowledge translation (KT) health-relevant retrievals (n=369) by type.

Resource type	Number of websites retrieved	Subset designated “educational in intent”
Journals or journal articles	115	1
Specific project or program descriptions	60	
Information about fee-based KT training opportunities (eg, Descriptions of KT curricula, training courses, conferences, events, face-to-face workshops, summits, and seminars)	40	
Online resources for doing KT (eg, guides, toolkits, templates, lists of links, or advisory services)	28	20
Books or book chapters	21	
Presentations (eg, PowerPoint or Prezi)	18	6
Standalone definitions of KT (the majority describing dissemination to researchers)	17	11
Hubs or networks for sharing research or practice ideas in a specific area of health care (eg, Communities of Practice)	11	1
KT grant information	10	
News items, media releases, or notices	9	
Blog posts mentioning KT	8	1
Unpublished reports	7	
Theses	6	
Conference papers	5	
Policy or position statements	4	
Databases	3	
Job advertisements	2	
Learning modules	2	2
Webinars	2	1
Clinical trials	1	
Total	369	43

To better understand some of the difficulties clinicians would face when searching for KT resources online, we performed some secondary analyses on the dataset retrieved. We first determined the range of different types of health resources retrieved by KT terms in open Web searching (Table 1).

We also categorised all Websites retrieved based on their preferred use of specific KT descriptors, bringing to the fore the distribution of KT synonyms across health and non-health fields (Table 2).

This shows that health websites were predominately retrieved by terms “knowledge translation” (24%), “research utilization/research utilisation” (24%), and “implementation science” (18%). They were rarely retrieved by terms “knowledge transfer” (2%) and “knowledge exchange” (5%).

Outside the health domain, we found the inverse. The most prevalent terms within the non-health sites retrieved were “knowledge exchange” (19%) and “knowledge transfer” (18%), with the least prevalent being “knowledge translation” (2%) and “implementation science” (7%).

Within the 592 non-health sites retrieved, some subject areas showed a stronger preference for specific KT terms than others (Table 3).

**Table 2:** Distribution of knowledge translation (KT) synonyms across health and non-health websites retrieved.

KT synonyms	Health & medicine websites retrieved (n=369)	Non-health websites retrieved (n=592)
Evidence-to-use	9%	12%
Implementation science	18%	7%
Knowledge exchange	5%	19%
Knowledge Translation	24%	2%
Knowledge-to-action	9%	12%
Knowledge transfer	2%	18%
Research into practice	10%	12%
Research utilization/research utilisation	24%	17%

**Table 3:** Non-health subject areas retrieved and their predominant terminology.

Non-health subject areas	Number of websites retrieved	KT terminology within subject areas	Prevalence of terminology across subject areas
Primary/secondary education	125	Research into practice	38%
		Research utilization	21%
Higher education	75	Research utilization	28%
		Knowledge exchange	27%
		Knowledge transfer	21%
Business and finance	60	Knowledge to action	37%
		Knowledge transfer	27%
Innovation/commercialization partnerships	35	Knowledge transfer	55%
		Knowledge exchange	40%
Environment and conservation	33	Knowledge to action	39%
		Knowledge exchange	33%
Social services (ie, disability, child welfare, social work)	32	Research utilization	44%
Technology	30	Knowledge transfer	37%
		Knowledge exchange	27%
Law	20	Evidence-to-use	55%
Public policy or policymaking	14	Evidence-to-use	36%

## Discussion

### Principal Findings

After reviewing an extensive number of websites retrieved by a wide range of KT terms, we were surprised to find so few examples of KT learning resources, either online or reported in the published literature. Despite a comprehensive search strategy, we only found one resource that fulfilled all our inclusion criteria. Even looking broader than health, we could not identify modules designed to improve understanding on the topic. We must therefore corroborate clinician accounts of a lack of free online KT learning opportunities.

The Web is not short on KT materials for interested clinicians to access. Many of these, such as those provided by CIHR, are of high quality. Arguably, however, these resources put the burden squarely on the clinician-learner to contextualize and interpret KT for real-world implementation. In our assessment, they also assume a certain level of prior knowledge and do not provide instructional scaffolding. Certainly the concepts within the materials we found are not organized in ways to make it easy for beginners to understand the key aspects of KT research and practice. Many resources do not define KT or else describe it in a way that makes it difficult to delineate its components. Given the difficult, sometimes esoteric arguments around deliberative change based on evidence of effectiveness, we view this as problematic for learners.

A further problem with many of the resources we reviewed is that they target one stakeholder group in the KT process (eg, researchers or policymakers) to the omission of others, or fail to clearly define the intended audience altogether. Furthermore, many resources exist as individual objects without integration into a design with an overarching theoretical framework. They also lack interactivity with no attempt to engage learners through self-reflection or self-assessment tasks.

There is also an existing accessibility issue where KT training is concerned. The majority of KT training opportunities we identified required face-to-face, multi-day, fee-based attendance, or involved a competitive admission process (Table 1). We also suspect many training opportunities lie behind the pay walls of online Learning Management Systems at universities where KT is taught as part of a curriculum. These modes of delivery will inevitably exclude the majority of the health workforce.

A further issue highlighted by this review is the difficulty surrounding KT information retrieval. Even experienced Web searchers may find it time-consuming to identify learning materials on KT given the large number of terms used to describe it, and the fact that many of these terms retrieve materials in non-health domains as diverse as education, business, environment, public administration, and law (Tables 2 and 3). Interestingly, we found some clear differences in term usage between health and non-health sites within our sample. It may be that within health the terms *knowledge translation* and *implementation science* are emergent frontrunners while other disciplines tend to favor alternative terms for describing similar processes and concepts.

KT searching is not helped by the inefficacy of Web searching in general. To achieve a minimal level of precision in our searching, we were required to forgo the simple Google search box for Google’s advanced search interface. We also used two different Web browsers, Mozilla and Chrome, and found that there were clear differences in what was retrieved by each browser, despite entering the same search in each. We also went much further than most searchers would in screening the results. As shown in Table 1, even health-related KT resources required extensive sifting to find actual training resources. Using terms indicative of education and learning, we still retrieved everything from journal articles, book chapters, advertisements for programs or grant opportunities, and even blog posts. We believe finding relevant education on a topic as important as this should not be so hard.

For clinicians, there is also the problem that the concept overlaps with other deliberative health care change processes such as quality improvement and guideline implementation. Clinicians may need KT training to disambiguate the many activities that form part of it (research creation, synthesis, dissemination, exchange, and transfer) and focus firmly on locally contextualized knowledge-practice gaps and ways to bridge them for optimized patient and health care outcomes.

### Limitations

Our investigation has several limitations. First, we did not use an exhaustive list of synonyms for KT. Terms such as *knowledge mobilisation* and *translational research,* for example, were not included and may have resulted in us overlooking appropriate resources. We may have also used rather narrow inclusion criteria where the concept of “interactivity” is concerned. Several of the resources we retrieved aimed at clinicians could be described as having an educative purpose. However, these same resources were excluded based on their design, rather than their content per se. A further limitation may be the use of a single, rather than dual, reviewing process when determining eligibility of each website. This was a pragmatic decision which may have resulted in some contestable exclusion decisions.

### Conclusions

Health care professionals have a stake in the widespread translation of well-designed research evidence into clinical practice. It is therefore important that they have access to opportunities to learn about KT and how it might drive improvements in the health outcomes of their patients. These learning opportunities should be available at times convenient to the clinician and would ideally present the complex concepts and processes associated with KT in a graduated and interactive way.

This review found only one Web-based resource that could be considered an interactive educational resource on KT for clinicians (Dementia KT). There is a need for more free online KT training resources targeted at clinicians that clearly define KT and its theories and methods, and help clinicians visualize how KT might work within their own local context.

## Appendix

Multimedia Appendix 1 Sample database search strategy.

## Abbreviations

AACODS: Authority, Accuracy, Coverage, Objectivity, Date, Significance
CIHR: Canadian Institutes of Health Research
DKT: Dementia Knowledge Translation
EBM: Evidence-Based Medicine
KT: knowledge translation
PRISMA: Preferred Reporting Items for Systematic Reviews and Meta-Analyses
