# Supplementary material for: How Do Clinicians Learn About Knowledge Translation? An Investigation of Current Web-Based Learning Opportunities
Source: JMIR Med Educ. 2017 Jul 13;3(2):e12. doi: 10.2196/mededu.7825 (PMC5532514; doi:10.2196/mededu.7825)
Supplement: Multimedia Appendix 1 [file mededu_v3i2e12_app1.pdf]

Sample database search strategy

Ovid MEDLINE(R) 1946 to Present

Searched 1 July 2015

#	Searches
1	(knowledge adj2 (translat* or transfer* or exchange)).tw,kw.
2	(Research utili?ation or implementation science or "research into practice" or "knowledge to action" or "evidence to use").tw,kw.
3	(PARiHS or "research implementation" or "research in practice").tw,kw.
4	or/1-3
5	education/ or exp curriculum/ or education, distance/ or education, professional/ or exp education, continuing/ or exp education, graduate/ or exp education, medical/ or exp education, nursing/ or exp education, pharmacy/ or education, public health professional/ or preceptorship/ or inservice training/ or staff development/ or exp teaching/
6	Learning/
7	ed.fs.
8	(educat* or curricul* or teach* or instruct* or learn* or train* or staff development or professional development or CPD or CME).tw,kw.
9	or/5-8
10	Computer-Assisted Instruction/ or Online Systems/ or Internet/ or Education, Distance/
11	Educational Technology/
12	((computer* or digital* or technolog* or electronic or online* or on-line or interactive or internet or web or distance or flexibl*) adj2 (class* or course* or educat* or instruct* or learn* or simulat* or train* or teach* or delivery or module* or tutorial*)).tw,kw.
13	(E-learn* or elearn* or mobile learn* or m-learn* or mlearn*).tw,kw.
14	(Massive Open Online Course? or MOOC?).tw,kw.
15	(Canvas network or Coursera or Coursesites or edx or Futurelearn or iversity or miriada x or moodle or novoed or openlearning or open2study or plato or spoc or udacity or pingpong).tw,kw.
16	Interactive tutorial/
17	Webcasts as topic/ or webcasts/
18	(Webcast* or webinar* or podcast*).tw,kw.
19	or/10-18
20	4 and 9 and 19
21	limit 20 to english language

Legend
adj = adjacency operator. Adj2 between two terms retrieves records where those terms appear with no more than two spaces (i.e. one word) between them. The words themselves can occur in any order.
tw = search on textwords (i.e. title and abstract terms)
kw = search on author-nominated keywords
/ = search on the Medical Subject Headings (MeSH) field

fs = search on a free-floating subheading
? = a point within or at the end of a word where the insertion of 0-1 letters is allowed.
* = truncation. Allows for variant endings to a word stem.
